# A Short-Term Exposure to Tributyltin Blocks Leydig Cell Regeneration in the Adult Rat Testis

**DOI:** 10.3389/fphar.2017.00704

**Published:** 2017-10-12

**Authors:** Xiaolong Wu, Jianpeng Liu, Yue Duan, Shiyu Gao, Yao Lü, Xiaoheng Li, Qiqi Zhu, Xianwu Chen, Jing Lin, Leping Ye, Ren-Shan Ge

**Affiliations:** ^1^Department of Anesthesiology, The Second Affiliated Hospital and Yuying Children’s Hospital of Wenzhou Medical University, Wenzhou, China; ^2^Center of Scientific Research, The Second Affiliated Hospital and Yuying Children’s Hospital of Wenzhou Medical University, Wenzhou, China; ^3^Department of Pediatric Pulmonology, The Second Affiliated Hospital and Yuying Children’s Hospital of Wenzhou Medical University, Wenzhou, China

**Keywords:** tributyltin, ethane dimethane sulfonate, Leydig cell, regeneration

## Abstract

**Background:** Tributyltin (TBT) is widely used as an antifouling agent that may cause reproductive toxicity. The mechanism of TBT on Leydig cell development is still unknown. The objective of the present study was to investigate whether a brief exposure to low doses of TBT permanently affects Leydig cell development and to clarify the underlying mechanism.

**Methods:** Adult male Sprague Dawley rats were randomly assigned into four groups and gavaged normal saline (control), 0.1, 1.0, or 10.0 mg/kg/day TBT for a consecutive 10 days, respectively. At the end of TBT treatment, all rats received a single intraperitoneal injection of 75 mg/kg ethane dimethane sulfonate (EDS) to eliminate all of adult Leydig cells. Leydig cells began a developmental regeneration process on post-EDS day 35. The Leydig cell regeneration was evaluated by measuring serum testosterone, luteinizing hormone, and follicle-stimulating hormone levels on post-EDS day 7, 35, and 56, the expression levels of Leydig cell genes, Leydig cell morphology and number and proliferation on post-EDS day 56.

**Results:** TBT significantly reduced serum testosterone levels on post-EDS day 35 and 56 and increased serum luteinizing hormone and follicle-stimulating hormone levels on post-EDS day 56 at ≥1 mg/kg/day. Immunohistochemical staining showed that there were fewer regenerated Leydig cells in the TBT-treated testis on post-EDS day 56. Further study demonstrated that the mRNA or protein levels of Leydig (*Lhcgr*, *Cyp11a1, Hsd3b1, Cyp17a1*, and *Hsd17b3*) and Sertoli cells (*Fshr*, *Dhh*, and *Sox9*) were significantly down-regulated in the TBT-treated testes when compared to the control. Immunofluorescent staining showed that TBT inhibited Leydig cell proliferation as judged by the reduced number of proliferating cyclin nuclear antigen-positive Leydig cells on post-EDS day 35.

**Conclusion:** The present study demonstrated that a short-term TBT exposure blocked Leydig cell developmental regeneration process via down-regulating steroidogenesis-related proteins and inhibiting the proliferation of Leydig cells.

## Introduction

Tributyltin chloride (TBT) has been extensively used in a variety of industrial products, as a wood preservative, biocide, and plastic stabilizer (Shawky and Emons, 1998). A widespread environmental contamination of TBT remains to date (Fent, 1996; Mitra et al., 2014). Human beings are exposed to TBT through the food ingestion (Mitra et al., 2014), dermal contact, and inhalation (Aluoch et al., 2007). TBT has been reported to cause the damage of many organs, including the testis. In the testis, the Leydig cell is also the target of TBT. In the isolated pig Leydig cells, TBT directly inhibited the last-step androgen biosynthetic enzyme, 17β-hydroxysteroid dehydrogenase 3 (HSD17B3, encoded by *Hsd17b3*) with the half maximal inhibitory concentration (IC_50_) of 114 nM (Ohno et al., 2005). TBT at 100 nM *in vitro* down-regulated the expression levels of cytochrome P450 17α-hydroxylase/20-lyase (CYP17A1, encoded by *Cyp17a1*) without affecting the expression levels of other steroidogenic enzymes such as cytochrome P450 cholesterol side chain cleavage enzyme (CYP11A1, encoded by *Cyp11a1*), 3β-hydroxysteroid dehydrogenase 1 (HSD3B1, encoded by *Hsd3b1*), and *Hsd17b3* as well as the cholesterol-transporting protein, the steroidogenic acute regulatory protein (StAR, encoded by *Star*) (Nakajima et al., 2005). Immature male mice after a single oral exposure to 25, 50, or 100 mg/kg TBT had the lower expression levels of *Cyp11a1*, *Hsd3b1*, *Cyp17a1*, and *Hsd17b3* in the testes at 50 and 100 mg/kg doses (Kim et al., 2008).

Leydig cells existing in the interstitial compartment of the testis are unique endocrine cells, primarily producing 95–99% of circulatory testosterone (Teerds et al., 2007). In the mature testis, a stable number of adult Leydig cells is maintained by a slow turn-over of Leydig cells via commitment of stem Leydig cells and their subsequent differentiation (Stanley et al., 2012). Interestingly, a rapid turn-over was achieved by a complete elimination after a single treatment of a chemical called ethane dimethane sulfonate (EDS) (Rommerts et al., 1988; Teerds et al., 1988; Vreeburg et al., 1988; Hu et al., 2010). Seven days after intraperitoneal injection of 75 mg/kg EDS to the adult rat, all of Leydig cells were eliminated, a developmental regeneration process began on post-EDS day 21 and completed on post-EDS day 56 to recover all of adult Leydig cells (Rommerts et al., 1988; Teerds et al., 1988; Vreeburg et al., 1988; Hu et al., 2010; Guo et al., 2013). Apparently, the developmental regeneration of Leydig cells was similar to the pubertal Leydig cell development with the appearance of progenitor Leydig cells on post-EDS day 21, differentiation into immature Leydig cells on post-EDS day 35, and maturation into adult Leydig cells on post-EDS day 56 (Guo et al., 2013; Zhang et al., 2015). This developmental regeneration process started from stem Leydig cells (Davidoff et al., 2004; Stanley et al., 2012). Therefore, it is a good model to study the effects of toxicants on the developmental process of Leydig cells in the adult testis. In the present study, we briefly exposed adult male rats to TBT for 10 days and then observed the impairment of Leydig cell developmental regeneration process later. The Leydig cell regeneration was evaluated by measuring serum testosterone, luteinizing hormone (LH), and follicle-stimulating hormone (FSH) levels on post-EDS day 7, 35, and 56, the expression levels of Leydig cell genes, Leydig cell morphology and number and proliferation on post-EDS day 56. We found that a short-term TBT exposure blocked Leydig cell developmental regeneration process via down-regulating steroidogenesis-related proteins and inhibiting the proliferation of Leydig cells, thus reducing testosterone levels.

## Materials and Methods

### Chemicals

TBT was obtained from Sigma-Aldrich (St. Louis, MO). SYBR Green qPCR Kit and BCA Protein Assay Kit was purchased from Takara (Otsu, Japan). Trizol was purchased from Invitrogen (Carlsbad, CA, United States). EDS was purchased from Pterosaur Biotech (Hangzhou, China). Immulite2000 Total Testosterone Kit was purchased from Sinopharm Group Medical Supply Chain Services Co., Ltd. (Hangzhou, Zhejiang, China). Radio immunoprecipitation assay (RIPA) buffer was obtained from Bocai Biotechnology (Shanghai, China).

### Animal Administration

Fifty-four 51-day-old male Sprague-Dawley rats (Laboratory Animal Center of Wenzhou Medical University, Wenzhou, China) were raised in a 12 h dark/light cycle temperature at 23 ± 2°C and relative humidity of 45–55%. Water and food were provided *ad libitum*. Rats were adjusted to the new environment after their shipping for a week before they were randomly divided into four groups (18 animals per group). The rats were housed in IVC cages (three rats per cage) on soft chip bedding and provided pellet chow (Shanghai Laboratory Animal Center). TBT dissolved in normal saline was gavaged to rats. Rats in group 1 orally received normal saline as the control, while rats in group 2, 3, and 4 orally received low doses of 0.1, 1.0, or 10.0 mg/kg/day TBT, respectively. The treatments were consecutively conducted for 10 days.

EDS was dissolved in a mixture of dimethyl sulfoxide and deionized sterile water (1:3, v/v). At the end of TBT treatment, all rats received a single intraperitoneal injection of 75 mg/kg EDS to eliminate all Leydig cells. Rats (six animals each group) were sacrificed on post-EDS day 7, 35, and 56 by asphyxiation with CO_2_. Trunk blood was collected, placed in a gel glass tube, and centrifuged at 1500 × *g* for 10 min to collect serum samples. Serum samples were labeled and stored at -80°C until hormone [testosterone, luteinizing hormone (LH), and follicle-stimulating hormone (FSH)] analysis. Furthermore, each pair of testes was separated and weighted. One testis each animal was frozen in the liquid nitrogen and stored at -80°C for subsequent gene and protein expression level analysis. The contralateral testis was punched three holes using a G27 needle and then fixed in Bouin’s solution for immunohistochemical analysis. All studies were approved by the Wenzhou Medical University’s Animal Care and Use Committee.

### RNA Isolation and Real-Time PCR (qPCR)

Total RNAs were purified from the testes using the Trizol Kit according to the manufacturer’s instructions, and the concentration of RNA was measured by reading OD value at 260 nm. The first strand (cDNA) was reversely transcribed and used as the template for qPCR analysis as previously described (Ge et al., 2005). The expression levels of Leydig (*Lhcgr*, *Scarb1*, *Star*, *Cyp11a1*, *Hsd3b1*, *Cyp17a1*, and *Hsd17b3*) and Sertoli cells (*Fshr, Amh, Dhh*, and *Sox9*) were measured using a SYBR Green qPCR Kit. The qPCR reaction mixture had 10 μl SYBR Green mix, 1.6 μl forward and reverse primer mix, 1 μg diluted cDNA sample, and 5–8 μl RNase-free water. The reaction was processed by the following program: 95°C for 5 min, followed by 40 cycles of 95°C for 10 s, and 60°C for 30 s. The Ct value was read and the expression level of a target gene was calculated using a standard curve method as previously described (Ge et al., 2005). The relative expression of testicular genes was normalized to *β-Actin* (*Actb*). The melting curve was examined for the quality of PCR amplification for each sample. The primers were listed in Supplementary Table S1.

### Immunohistochemical Staining and Cell Counting

We used HSD3B1 as the biomarker of all Leydig cells and 11β-hydroxysteroid dehydrogenase 1 (HSD11B1) as the Leydig cells at the advanced stage (Guo et al., 2013), and SOX9 as the biomarker of Sertoli cells (Koopman, 1999). To enumerate number of HSD3B1-positive Leydig cells and SOX9-positive Sertoli cells, sampling of the testis was performed according to a fractionator technique as previously described (Mendis-Handagama et al., 1989). Briefly, six testes per group collected at each time point were randomly selected. Each testis was cut in eight parts as disks and two parts were randomly selected. Then, the two parts were cut into four pieces and one piece was randomly selected from total 8 pieces. The piece per testis was embedded in paraffin in a tissue array. Paraffin block was sectioned in 6-μm-thick sections. Approximately, ten sections were randomly selected from each testis per rat. Sections were used for immunohistochemical staining. Avidin-biotin immunohistochemical staining was performed according to the manufacturer’s instructions (Vector, Burlingame, CA, United States). Microwave heating in a citrate buffer (10 mM, pH 6.0) for 10 min was used for the antigen retrieval. The endogenous peroxidase was blocked with 0.5% of H_2_O_2_ in methanol for 30 min. Sections were incubated with an HSD3B1, HSD11B1 or SOX9 antibody (1:1000 dilution, v/v) for 1 h at room temperature. The antibody-antigen complexes were visualized with diaminobenzidine as the brown cytoplasmic staining for positively labeled Leydig cells and the brown nuclear staining for positively labeled Sertoli cells. The sections were counterstained with Mayer hematoxylin, dehydrated in graded concentrations of ethanol and cover-slipped with resin. Images were taken and total microscopic fields per section were counted. The total number of Leydig cells was calculated by multiplying the number of Leydig or Sertoli cells counted in a known fraction of the testis by the inverse of the sampling probability.

### Computer-Assisted Image Analysis of Cell Size and Nuclear Size

Leydig cells were identified by staining HSD3B1 as above. The Leydig cell size, nuclear size, and cytoplasmic size were calculated as previously described (Liu et al., 2016). Five randomly selected fields in each of three non-adjacent sections per testis were captured using a BX53 Olympus microscope (Tokyo, Japan) equipped with digital camera interfaced to a computer. The images that were displayed on the monitor represented partial area of a testis. Cell size and nuclear size were estimated using the image analysis software (Image-Pro Plus; Media Cybernetics, Silver Spring, MD, United States). More than 50 Leydig cells were evaluated in each testis. The cell size and nuclear size and cytoplasmic size were calculated.

### Semi-Quantitative Immunohistochemical Measurement of HSD11B1 and SOX9

HSD3B1 is a protein in Leydig cells (Ge and Hardy, 1998). SOX9 is a transcription factor in Sertoli cells for its function (Koopman, 1999). Immunohistochemical staining of HSD3B1 and SOX9 was performed as stated above. Target protein density and background area density were measured using the image analysis software (Image-Pro Plus; Media Cybernetics, Silver Spring, MD, United States) according to the manufacturer’s instruction. More than 50 Leydig or Sertoli cells were evaluated in each testis and the density of each sample was averaged.

### Calculation of Leydig Cell Proliferation

The Leydig cell proliferation was judged by immunofluorescent staining of PCNA after the dual staining of PCNA (for proliferating cell) and HSD11B1 (the Leydig cells) in testis collected on post-EDS day 35, when the regenerated Leydig cells were at the stage of immature Leydig cell, with proliferating capacity (Guo et al., 2013). The sections in the tissue array assembled above were used. Sections were sequentially incubated with the primary antibodies of HSD11B1 and PCNA for 60 min. Then, the fluorescent secondary antibody (Alexa-conjugated anti-rabbit or anti-mouse IgG, 1:500) was used to label Leydig cells (HSD11B1, cytoplasmic staining in green color) and proliferating cells (PCNA, nuclear staining in red color). Images were taken with a fluorescent microscopy.

### Western Blot

Testes were homogenized in the ice-cold PBS and then the homogenates were lysed with RIPA buffer to obtain proteins. The protein concentrations of samples were measured using BCA Protein Assay Kit in a plate reader. An aliquot of 30 μg protein each sample was electrophoresed in 10% polyacrylamide gels containing sodium dodecyl sulfate and then the separated proteins were electrically transferred onto the nitrocellulose membranes. The membranes were blocked with 5% non-fat milk in Tris-buffered saline Tween-20 buffer for 1 h. Then, the membranes were incubated with primary antibodies against LHCGR, SCARB1, CYP11A1, HSD3B1, CYP17A1, HSD11B1, FSHR, AMH, DHH, SOX9, and ACTB (listed in Supplementary Table S2) overnight at 4°C. The membranes were then washed and incubated with HRP-conjugated anti-rabbit or anti-goat IgG secondary antibody (1:5000, Bioword, United States) for 1 h at room temperature and washed three times. The immunoreactive bands were visualized by chemiluminescence using an ECL kit (Amersham, Arlington Heights, IL, United States). The intensity of band was analyzed with Image Lab software.

### Statistical Analysis

All data are presented as the mean ± standard errors (SEM). Statistical significance was analyzed using one-way ANOVA followed by ad hoc Turkey’s multiple comparisons to the control. Statistical analysis was performed using GraphPad Prism (version 6, GraphPad Software Inc., San Diego, CA, United States). A *p* < 0.05 was considered statistically significant.

## Results

### General Parameters of TBT Toxicity

To analyze the general parameters of TBT toxicity, body weights were recorded before or at the end of TBT treatment as well as on post-EDS day 7, 35, and 56 (**Table 1**). Testis weights were recorded on post-EDS day 7, 35, and 56. TBT did not affect body and testis weights before post-EDS day 56 (Table 1). Interestingly, the body weight in 10 mg/kg TBT group was significantly lower than that of the control on post-EDS day 56. No mortalities and abnormal activities were observed in rats of any groups.

**Table 1:** General parameters of toxicology after treatment of tributyltin.

Parameters	Dosage (mg/kg)			
	0	0.1	1	10
**Body weight**				
Before TBT treatment	288.5 ± 8.22	281.6 ± 6.91	284.5 ± 5.83	287.9 ± 5.60
After TBT treatment	337.8 ± 19.1	315.25 ± 25.3	309.3 ± 51.7	302.5 ± 18.0
Post-EDS day 7	320.0 ± 15.90	306 ± 20.45	301.6 ± 11.40	291.6 ± 16.89
Post-EDS day 35	419.6 ± 28.51	428.6 ± 49.39	434.0 ± 27.14	377.0 ± 37.80
Post-EDS day 56	522.2 ± 29.74	485.2 ± 45.78	460.0 ± 56.06	439.4 ± 36.50*
**Testes weight**				
Post-EDS day 7	1.28 ± 0.35	1.32 ± 0.35	1.30 ± 0.21	1.27 ± 0.18
Post-EDS day 35	1.36 ± 0.35	1.37 ± 0.30	1.41 ± 0.19	1.42 ± 0.05
Post-EDS day 56	2.17 ± 0.19	2.08 ± 0.23	1.89 ± 0.15	1.87 ± 0.10


*Mean ± SEM, n = 6. ^∗^P < 0.05 indicates significant difference when compared to the control (0 mg/kg) at each time point*.

### TBT Lowers Testosterone Levels

**FIGURE 1:**
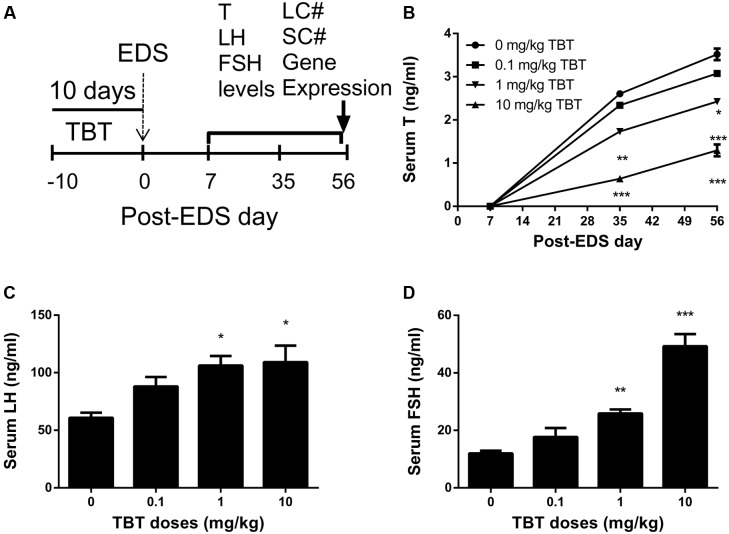
Serum levels of testosterone (T), luteinizing hormone (LH), and follicle-stimulating hormone (FSH) after tributyltin (TBT) exposure. **(A)** Experimental plan; **(B)** Serum T levels on post-EDS (ethane dimethane sulfonate) day 7, 35, and 56; **(C)** Serum LH levels on post-EDS day 56; **(D)** Serum FSH levels on post-EDS day 56. Mean ± SEM, *n* = 6. ^∗^, ^∗∗^, ^∗∗∗^ indicate significant difference when compared to the control (CON) at *p* < 0.05, 0.01, and 0.001, respectively.

Sera were collected on post-EDS day 7, 35, and 56 for hormone (testosterone, LH, FSH) analysis (**Figure 1**). Serum testosterone levels in all groups were undetectable on post-EDS day 7, confirming that all Leydig cells were completely killed by EDS (Guo et al., 2013). Testosterone levels in all groups were gradually elevated on post-EDS day 35, confirming that Leydig cell developmental regeneration process progressed (Guo et al., 2013). TBT dose-dependently decreased testosterone levels with significant difference being observed at ≥1 mg/kg on post-EDS day 35 and at ≥0.1 mg/kg on post-EDS day 56 (**Figure 1B**). The results suggest that TBT delays Leydig cell developmental regeneration. Further analysis showed that TBT significantly increased serum LH and FSH levels at ≥1 mg/kg on post-EDS day 56 (**Figures 1C,D**), indicating that both Leydig cell function and spermatogenesis are damaged.

### TBT Reduces Leydig Cell Number

**FIGURE 2:**
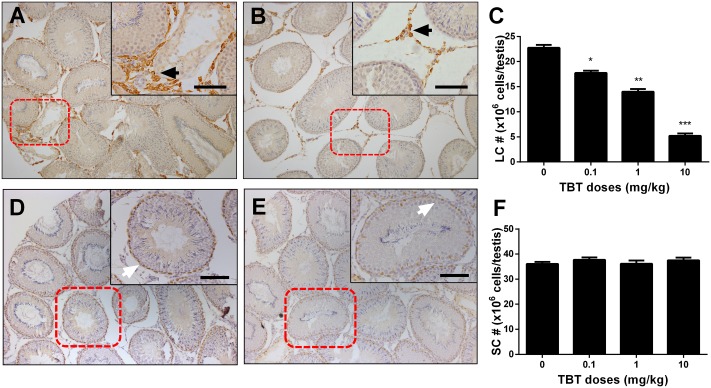
The effects of tributyltin (TBT) on Leydig cell (LC) and Sertoli cell (SC) number. Leydig cells were identified by immunohistochemical staining of 3β-hydroxysteroid dehydrogenase 1 (HSD3B1) and Sertoli cells were identified by staining of SOX9 in rat testis sections from post-EDS day 56 and enumerated by the stereological method. Images of HSD3B1 staining (with inserts): **(A)** control; **(B)** TBT 10.0 mg/kg dose. Bar = 50 μm. Black arrow points to Leydig cells. **(C)**: quantification of Leydig cell (LC) number (#). Images of SOX9 staining (with inserts): **(D)** control; **(E)** TBT 10.0 mg/kg dose. Bar = 50 μm. White arrow points to Sertoli cells. **(F)**: quantification of Sertoli cell (SC) number (#). Mean ± SEM, *n* = 6. ^∗^, ^∗∗^, ^∗∗∗^ indicate significant difference when compared to the control (TBT 0 mg/kg) at *p* < 0.05, 0.01, and 0.001, respectively.

Leydig cells were stained by the biomarker, HSD3B1, on post-EDS day 56. Sertoli cells were stained by the biomarker, SOX9. When compared to the control, TBT dose-dependently decreased Leydig cell number (**Figures 2A–C**). However, Sertoli cell number did not change (**Figures 2D–F**). This indicates that TBT delays Leydig cell developmental regeneration process.

### TBT Down-Regulates Leydig and Sertoli Cell Gene Expressions

**FIGURE 3:**
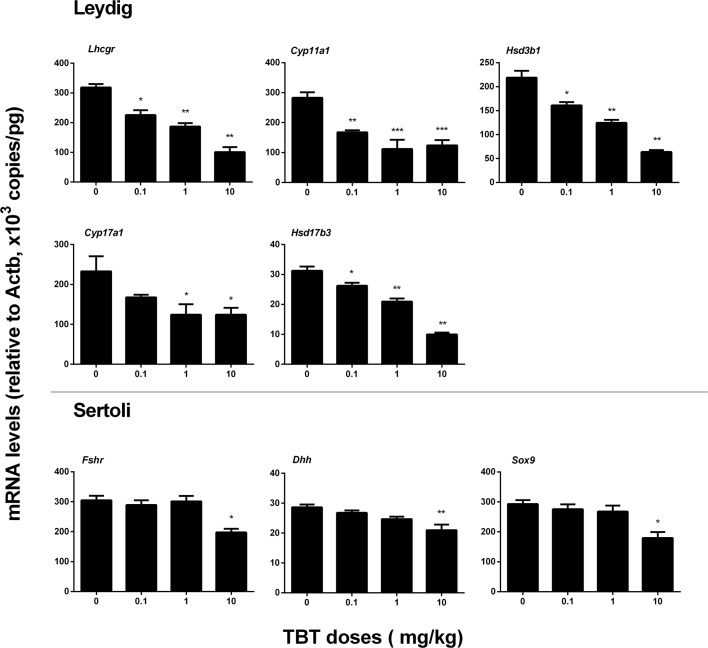
Expression levels of Leydig and Sertoli cell genes in the tributyltin (TBT)-treated testis on the post-EDS day 56. Leydig cell genes: *Lhcgr*, *Cyp11a1*, *Hsd3b1*, *Cyp17a1*, and *Hsd17b3.* Sertoli cell genes: *Fshr*, *Dhh*, and *Sox9*. Mean ± SEM, *n* = 6. ^∗^, ^∗∗^, ^∗∗∗^ indicate significant difference when compared to the control (TBT 0 mg/kg) at *p* < 0.05, 0.01, and 0.001, respectively.

We measured the expression levels of Leydig (*Lhcgr*, *Scarb1*, *Star*, *Cyp11a1*, *Hsd3b1*, *Cyp17a1*, and *Hsd17b3*) and Sertoli (*Fshr*, *Amh*, *Dhh*, and *Sox9*) cell genes on post-EDS day 56. TBT dose-dependently down-regulated the expression levels of Leydig cell genes, including *Lhcgr*, *Cyp11a1*, *Hsd3b1*, and *Hsd17b3* at ≥0.1 mg/kg and *Cyp17a1* at ≥1 mg/kg without affecting the levels of *Scarb1* and *Star* (**Figure 3**). TBT at 10 mg/kg also down-regulated *Fshr*, *Dhh*, and *Sox9* gene expression levels in Sertoli cells while lower doses (0.1 and 1 mg/kg) of TBT did not affect the expression levels of these genes (**Figure 3**). These results suggest that both Leydig and Sertoli cells are affected by TBT and Leydig cells are more sensitive to TBT insult.

### TBT Reduces Protein Expression Levels in Leydig and Sertoli Cells

**FIGURE 4:**
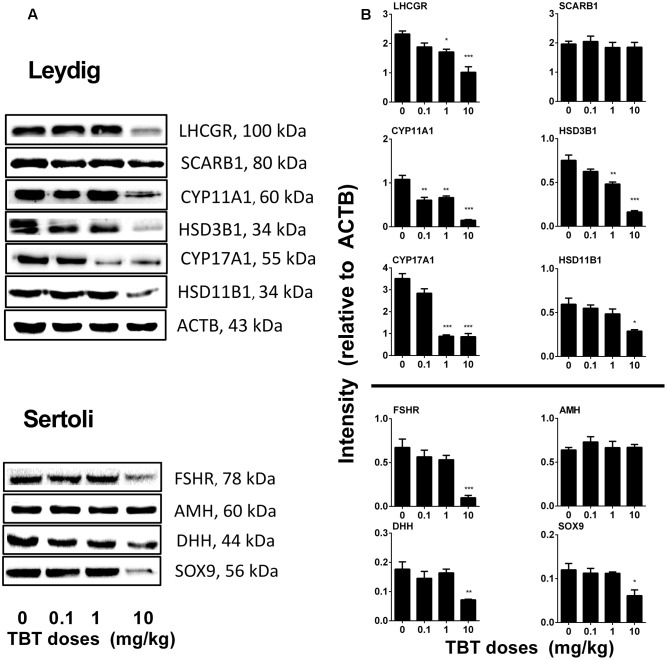
Expression levels of Leydig and Sertoli cell gene products in the tributyltin (TBT)-treated testis on the post-EDS day 56. **(A)**:Gel images; **(B)**: quantitative result. Leydig cell proteins: LHCGR, SCARB1, CYP11A1, HSD3B1, CYP17A1, and HSD11B1. Sertoli cell proteins: FSHR, AMH, DHH, and SOX9. Mean ± SEM, *n* = 3. ^∗,^^∗∗,^^∗∗∗^ indicate significant difference when compared to the control (TBT 0 mg/kg) at *p* < 0.05, 0.01, and 0.001, respectively.

**FIGURE 5:**
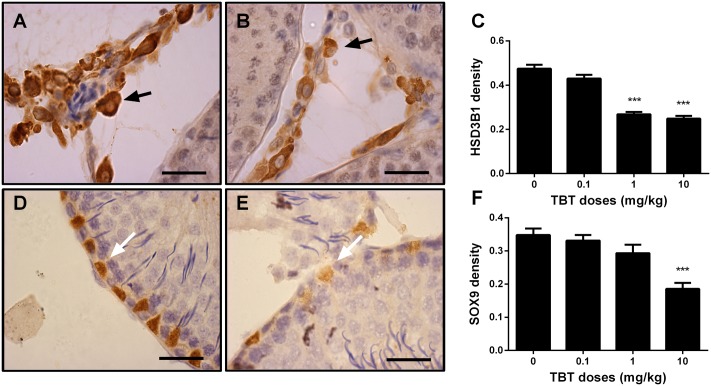
The semi-quantitative assay of 3β-hydroxysteroid dehydrogenase 1 (HSD3B1) and SOX9 in rat testis sections from post-EDS day 56. Images of HSD3B1 staining: **(A)** control; **(B)** TBT 10.0 mg/kg dose. Images of SOX staining: **(D)** control; **(E)** TBT 10.0 mg/kg dose. Black arrow points to HSD3B1 staining (cytoplasm); and white arrow points to SOX9 staining (nucleus). **(C,F)**: quantification of density of HSD3B1 and SOX9, respectively. Mean ± SEM, *n* = 6. ^∗^, ^∗∗^, ^∗∗∗^ indicate significant difference when compared to the control (TBT 0 mg/kg) at *p* < 0.05, 0.01, and 0.001, respectively.

We measured the expression levels of Leydig (LHCGR, SCARB1, CYP11A1, HSD3B1, CYP17A1, and HSD11B1) and Sertoli (FSHR, AMH, DHH, and SOX9) cell proteins on post-EDS day 56. TBT lowered these protein levels in parallel with their mRNA expression levels (**Figure 4**). We further used a semi-quantitative analysis of HSD3B1 (Leydig cell biomarker) and SOX9 (Sertoli cell biomarker) densities in the individual cell and found that TBT lowered HSD3B1 level at ≥1 mg/kg and SOX9 level at 10 mg/kg (**Figure 5**). These results suggest that TBT impaired Leydig functions at both low and high doses but Sertoli cell functions only at high dose.

### TBT Reduces Leydig Cell Size and Cytoplasmic Size

**FIGURE 6:**
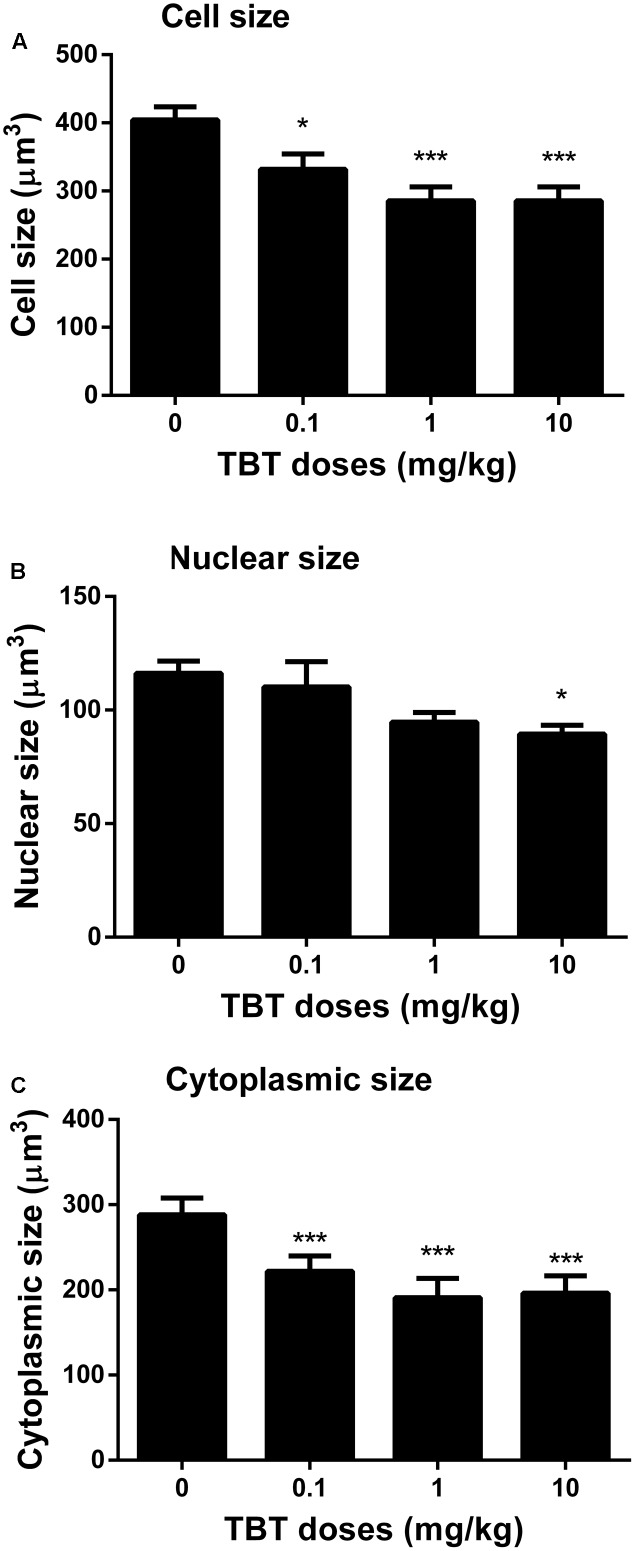
The Leydig cell size, nuclear size, and cytoplasmic size in rat testis sections from post-EDS day 56. **(A)** Leydig cell size; **(B)** Leydig cell nuclear size; **(C)** Leydig cell cytoplasmic size. Mean ± SEM, *n* = 6. ^∗^, ^∗∗^, ^∗∗∗^ indicate significant difference when compared to the control (TBT 0 mg/kg) at *p* < 0.05, 0.01, and 0.001, respectively.

We measured cell size and cytoplasmic size of Leydig cells in the testis on post-EDS day 56 (**Figure 6**). TBT reduced Leydig cell size and cytoplasmic size at ≥0.1 mg/kg although it also reduced the nuclear size of Leydig cells at 10 mg/kg, indicating that Leydig cells after exposure to TBT are less mature than those in the control.

### TBT Lowers Proliferation of Immature Leydig Cells

**FIGURE 7:**
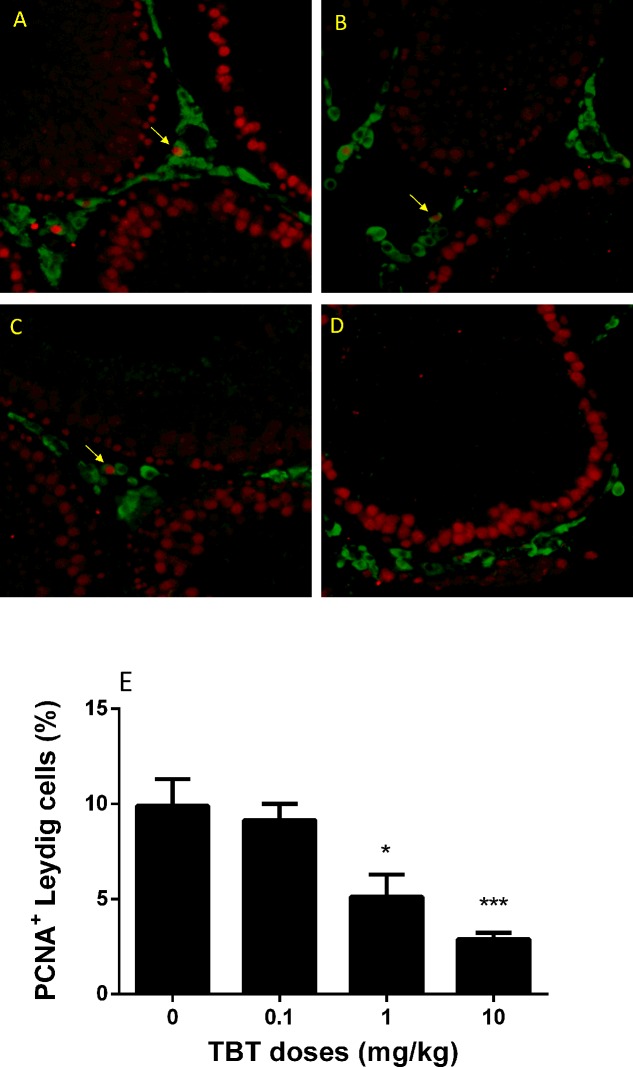
The percentage of PCNA (proliferating)-positive and 11β-hydroxysteroid dehydrogenase 1 (HSD11B1, Leydig cell)-positive cells in rat testis sections from post-EDS day 56. Images: **(A)** control; **(B)** TBT 0.1 mg/kg dose; **(C)** TBT 1 mg/kg dose; **(D)** TBT 10 mg/kg dose. PCNA = red color in the nucleus; HSD11B1 = green color in the cytoplasm. **(E)** Quantification of data for the percentage of PCNA*^+^*-HSD11B1*^+^* cells in all HSD11B1*^+^* cells. Mean ± SEM, *n* = 6. ^∗^, ^∗∗^, ^∗∗∗^ indicate significant difference when compared to the control (TBT 0 mg/kg) at *p* < 0.05, 0.01, and 0.001, respectively.

Immature Leydig cells on post-EDS day 35 have the capacity of proliferation (Guo et al., 2013). PCNA is a well-known nuclear matrix protein for cell proliferation. We stained Leydig cells using HSD11B1 and the proliferating cell using PCNA. There were fewer PCNA-positive immature Leydig cells in TBT-treated testis sections at ≥1 mg/kg TBT groups when compared to the control (**Figure 7**), indicating that TBT inhibits immature Leydig cell proliferation.

## Discussion

In the present study, we used an EDS-induced rat Leydig cell regeneration model, which is very unique to study the developmental process of Leydig cells in the adult rat testis. Adult Leydig cells in rat testis are depleted by i.p. 75 mg/kg EDS (Teerds, 1996). The elimination of Leydig cells caused the increased secretion of LH and production of local growth factors and cytokines, which induced the Leydig cell developmental regeneration process (Kerr and Sharpe, 1985; Teerds, 1996). Like the developmental process of Leydig cells during the puberty, the developmental regeneration process in adult rat testis undergoes three distinct stages: the commitment of stem into progenitor Leydig cells by post-EDS day 21, and then the differentiation into immature Leydig cells by post-EDS day 28, and final maturation into adult Leydig cells by day 56 (Guo et al., 2013). Herein, we adopted this model to test whether TBT disrupted Leydig cell developmental process.

After oral administration, TBT can be readily absorbed and distributed in many tissues and it is also metabolized to dibutyltin and monobutyltin. Pregnant dams were gavaged TBT (10 mg/kg) from GD 8 to postnatal day 12, and TBT levels in dam’s serum, placenta, liver, and brain could reach about 150 ng/ml, 750 ng/g, 1500 ng/ml, and 1750 ng/ml on GD20, respectively (Cooke et al., 2008). This suggests that TBT can enter many tissues.

A short-term period (10 days) of the TBT exposure appeared to disrupt Leydig cell developmental process permanently. Firstly, TBT exposure lowered serum testosterone levels following the treatment of EDS even at as low as 0.1 mg/kg. Secondly, the mRNA levels of Leydig cell steroidogenesis-related genes and their protein products were down-regulated in TBT-exposed animals. These mRNAs include *Lhcgr, Cyp11a1, Hsd3b1, Cyp17a1*, and *Hsd17b3*. Thirdly, TBT significantly decreased HSD3B1-positive Leydig cell number regenerated after EDS treatment.

Although the exact time-point for TBT action on cell proliferation is still unknown, the decreased proliferating capacity of immature Leydig cells on post-EDS 35 was observed, as judged by the lower number of PCNA-positive immature Leydig cells. The reduction of Leydig cell numbers accounted for the reductions in both the serum testosterone levels and the expressions of the critical steroidogenic enzymes. However, the quality of Leydig cells (the capability of secreting testosterone *per se*) seemed also affected after TBT. In this regard, the reduction of Leydig cell size and cytoplasmic size and decrease in the HSD3B1 density per Leydig cell were observed, indicating that these regenerated Leydig cells are less mature.

A short-term period (10 days) of TBT exposure may disrupt Leydig cell developmental process by different mechanisms: directly acting on precursor or developing Leydig cell themselves, or indirectly by affecting the niche that regulates Leydig cell development. The evidence obtained herein indicates that the Leydig cell lineage was the primary target and that the late (via Sertoli cells) may play a role at the higher doses. A brief period (10 days) of TBT at the dose of 10 mg/kg seemed permanently disrupted Sertoli cell function, as judged by the reduced expressions of FSHR, DHH, and SOX9. SOX9 is the critical transcription factor for Sertoli cell development and maturation (Foster, 1996; Barrionuevo et al., 2006). The impairment of Sertoli cell functions was reflected by the elevation of FSH level via the negative feed-back mechanism.

Several studies regarding the effects of TBT on male reproductive functions have been conducted. When male Wistar rats orally received 0.5 or 15 mg TBT from postnatal day 23–53, 15 mg/kg TBT delayed the completion of preputial separation, lowered testosterone and LH levels (Grote et al., 2004). Male mice were gavaged a single dose of 25, 50, or 100 mg/kg of TBT lowered serum testosterone levels and down-regulated expression levels of *Cyp11a1*, *Cyp17a1*, *Hsd3b*, and *Hsd17b3*, although these doses were much higher than those used in the present study. However, the present study we used a completely different administration regimen of TBT, during which the regenerated cells in the Leydig cell lineage may be not exposed and only the stem Leydig cell and its niche was exposed to TBT. In the regard, the direct effect of TBT on LH secretion (lower LH levels) in the present study was not observed as in the previous study (Grote et al., 2004). On the contrary, LH levels on post-EDS day 56 were dose-dependently increased after TBT treatment, possibly via a negative feed-back action.

## Conclusion

A short-term exposure to low dose of TBT can significantly disrupt Leydig cell developmental process in adult animals. Its effect seems most like through directly disrupting stem and precursor Leydig cells. At the higher dose, the Sertoli cell function was also damaged, thus indirectly affecting Leydig cell development.

## Author Contributions

XW, LY, and R-SG have conceptualized the study design; XW, JpL, YD, SG, YL, XL, QZ, and XC have performed the experiments and collected the data; XW, JiL and R-SG have analyzed the data; XW, LY, and R-SG have written the manuscript.

## Conflict of Interest Statement

The authors declare that the research was conducted in the absence of any commercial or financial relationships that could be construed as a potential conflict of interest.
