# Supplementary material for: A Short-Term Exposure to Tributyltin Blocks Leydig Cell Regeneration in the Adult Rat Testis
Source: Front Pharmacol. 2017 Oct 12;8:704. doi: 10.3389/fphar.2017.00704 (PMC5643909; doi:10.3389/fphar.2017.00704)
Supplement: Supplementary file 1 [file Table_1.DOC]

**Supplementary Table S1. Primer information**

| **Primer**  **Symbol** | **Gene name** | **Primer direction** | **Sequences (5’to 3’)** | **PCR**  **(bp)** | **Accession** |
| --- | --- | --- | --- | --- | --- |
| Lhcgr | Luteinizing hormone receptor | Forward | CTGCGCTGTCCTGGCC | 103 | NM_012978 |
|  |  | Reverse | CGACCTCATTAAGTCCCCTGAA |  |  |
| Scarb1 | Scavenger receptor class B, member 1 | Forward | ATGGTACTGCCGGGCAGAT | 117 | NM_031541 |
|  |  | Reverse | CGAACACCCTTGATTCCTGGTA |  |  |
| Star | Steroidogenic acute regulatory protein | Forward | CCCAAATGTCAAGGAAATCA | 187 | NM_031558 |
|  |  | Reverse | AGGCATCTCCCCAAAGTG |  |  |
| Cyp11a1 | Cholesterol side chain cleavage enzyme | Forward | AAGTATCCGTGATGTGGG | 127 | NM_017286 |
|  |  | Reverse | TCATACAGTGTCGCCTTTTCT |  |  |
| Hsd3b1 | 3β-Hydroxysteroid dehydrogenase 1 | Forward | CCCTGCTCTACTGGCTTGC | 189 | NM_001007719 |
|  |  | Reverse | TCTGCTTGGCTTCCTCCC |  |  |
| Cyp17a1 | P450 17α-hydroxylase/ 17,20-lyase | Forward | TGGCTTTCCTGGTGCACAATC | 90 | NM_012753 |
|  |  | Reverse | TGAAAGTTGGTGTTCGGCTGAAG |  |  |
| Hsd17b3 | 17β-Hydroxysteroid dehydrogenase 3 | Forward | TGAAAGTTGGTGTTCGGCTGAAG | 202 | NM_054007 |
|  |  | Reverse | TGAAAGTTGGTGTTCGGCTGAAG |  |  |
| Fshr | Follicle stimulating hormone receptor | Forward | CCACAAGCCAATACAAACTAACT | 327 | NM_199237 |
|  |  | Reverse | CAAAAGTCCAGCCCAATACC |  |  |
| Amh | Antimulerian hormone | Forward | GCCCTAACCCTTCAACCA | 82 | NM_012902 |
|  |  | Reverse | GGGAATCAGAGCCAAACAGA |  |  |
| Dhh | Desert Hedgehog | Forward | AACCCCGACATAATCTTCA | 150 | NM_053367 |
|  |  | Reverse | CTCGTCCCAACCTTCAGT |  |  |
| Sox9 | SRY box 9 | Forward  Reverse | TGCTGAACGAGAGCGAGAAG  ATGTGAGTCTGTTCGGTGGC | 160 | NM_080403.1  NM_001141945 |
| β-actin | Beta-Actin | Forward | CCATGAAGATCAAGATCAT | 106 |  |
|  |  | Reverse | TTGCTGATCCACATCTGCT |  |  |
