# Supplementary material for: A Short-Term Exposure to Tributyltin Blocks Leydig Cell Regeneration in the Adult Rat Testis
Source: Front Pharmacol. 2017 Oct 12;8:704. doi: 10.3389/fphar.2017.00704 (PMC5643909; doi:10.3389/fphar.2017.00704)
Supplement: Supplementary file 2 [file Table_2.DOC]

**Supplementary Table 2. Antibodies**

| **Antibody** | **Species** | **Vendor (City, State, catalogue)** | **Dilution** | |
| --- | --- | --- | --- | --- |
|  |  |  | **WB** | **HS** |
| β-actin | rabbit | Cell Signaling Technology (Danvers,  MA,12620) | 1:1000 | ND |
| LHCGR | goat | Santa Cruz (Santa Cruz, CA,  sc-26343) | 1:1000 | ND |
| CYP11A1 | rabbit | Santa Cruz (Santa Cruz, CA,  sc-18043) | 1:1000 | ND |
| 3β-HSD1 | rabbit | Abcam (San Francisco, CA,ab65156) | 1:2000 | 1:1000 |
| CYP17A1 | rabbit | Santa Cruz (Santa Cruz, CA,  [sc-66850](https://www.scbt.com/scbt/product/cyp17a1-antibody-m-80?requestFrom=search)) | 1:1000 | ND |
| 11β-HSD1 | rabbit | Abcam (San Francisco, CA,  ab39364) | 1:2000 | 1:1000 |
| FSHR | rabbit | Abcam (San Francisco, CA,ab103874) | 1:2000 | ND |
| DHH | rabbit | Proteintech (Rosemont, IL,  13889-1-AP) | 1:2000 | ND |
| AMH | rabbit | Abcam (San Francisco, CA, ab84952) | 1:2000 | ND |
| SOX9 | rabbit | Abcam (San Francisco, CA,ab3697) | 1:2000 | 1:1000 |
| SCARB1 | rabbit | Abcam (San Francisco, CA, ab52629) | 1:1000 | ND |
| PCNA | mouse | Abcam (San Francisco, CA, ab29) | ND | 1:500 |

ND = Not detected; WB = Western blot; HS = Histochemical staining.
